# The effect of narrowband ultraviolet B phototherapy on serum folate level

**DOI:** 10.22088/cjim.12.2.180

**Published:** 2021-03

**Authors:** Azar Shirzadian Kebria, Meghdad Hosseini, Sorayya Khafri

**Affiliations:** 1 Department of Dermatology, Yahyanejad Hospital, Babol University of Medical Sciences, Babol, Iran; 2 Infertility and Reproductive Health Research Center, Health Research Institute, Babol University of Medical Sciences, Babol, Iran

**Keywords:** Diabetic peripheral neuropathy, Nortriptyline, Duloxetine, Diabetes, Pain

## Abstract

**Background:**

Narrowband ultraviolet B (NB-UVB) phototherapy has been used as a common treatment for dermatologic diseases such as psoriasis and vitiligo and generally considered a safe form of therapy during pregnancy. Invitro photodegradation of folate after exposure to UVB radiation has been documented but studies on UVB-induced alternation of serum folate level have reported inconsistent results. The aim of this study was to investigate the effect of NB-UVB radiation on serum folate level.

**Methods:**

In this study, serum folate levels were evaluated in patients at baseline and after 30 sessions of NB-UVB irradiation.

**Results:**

Twenty patients completed the study: 10 psoriasis, 7 vitiligo and 3 mycosis fungoides (patch stage). Mean serum folate level had significantly decreased from 2.76±0.59 ng/ml at baseline to 1.34±0.15 ng/ml after 30 sessions. (Mean NB-UVB cumulative dose 40.35±16.80 j/cm^2^, P=0.001).

**Conclusion:**

Serum folate levels may decrease after long-term NB-UVB phototherapy in patients with skin disorders.

NB-UVB phototherapy is commonly used for the treatment of various skin disorders including psoriasis, vitiligo, atopic dermatitis and early stage mycosis fungoides (MF) (1, 2). This therapeutic modality is generally considered by dermatologists as safe in pregnant women (3). Photolysis of folate has been documented especially when serum is exposed in vitro to UVA radiation (4). Some studies have suggested that NB-UVB phototherapy can diminish folate in humans (5, 6), while other studies have shown that folate level is not affected by NB-UVB phototherapy (7-9). In humans, folate deficiency has been connected with megaloblastic anemia, neural tube defects (NTDs) in the neonate, neuron psychiatric disorders and cardiovascular disease. It has been also involved in the development of colorectal carcinoma (10). Folate photodegradation after NB-UVB exposure seems to be important in patients with psoriasis or vitiligo. In patients with psoriasis, folate levels may be lower than in normal controls (11). This may be due to increased utilization of folic acid by the skin epidermal cells as a result of the rapid turnover (12). In comparison with the normal populations, patients with vitiligo often show decreased serum levels of folic acid (13). Folate deficiency results in hyperhomocysteinemia that may play a role in the destruction of melanocytes via increased oxidative damage (14, 15). Folate photodegradation in these patients may aggravate complications associated with folate deficiency. The aim of the present study was to investigate the effect of NB-UVB phototherapy on serum folate level in patients with dermatologic diseases.

## Methods

This study was carried out in the phototherapy unit of the dermatology department at Babol Medical University Hospital. Thirty patients undergoing NB-UVB phototherapy were enrolled from Mars 2015 to Aug 2016 in this study. All patients signed an informed consent form. The exclusion criteria for the studied subjects included the current use of folate supplements, chronic hepatic or renal diseases, taking drugs that interfere with folate metabolism including methotrexate, antiepileptics and any form of phototherapy three months before the study.

Ten patients did not complete treatment sessions and were excluded; thus, the study was performed on twenty patients: 10 psoriasis, 7 vitiligo and 3 mycosis fungoides (patch stage). Mean age was 35.8 years (range 11-56) and there was an equal number of males and females. Sixteen patients had skin photo type III, two patients had skin phototype II and two patients had skin phototype IV.

NB-UVB irradiation of the whole body was performed using a Daavlin cabinet (Daavlin, Bryan, OH, USA) equipped with 48 fluorescent lamp (Philips TL 01-100W UVB Narrowband,311nm). The patients received 2-3 treatments weekly starting with an initial UVB doses ranged from 0.14 to 0.3 J/cm^2^ which a mounts to about 70% MED (minimal erythema dose) with an increase by approximately 10-20% of the previous well-tolerated dose up to a maximum dose of 3.6 J/cm^2^. Serum folate was measured in all patients at the beginning of phototherapy and after 30 NB-UVB exposures using an Enzyme Immunoassay method (Monobind, USA) in the Biochemistry department of Babol Medical University. In accordance with this assay, normal range of serum folate is 2.5-13 ng/ml. software (SPSS, Version 21) was used to analyze the results. Wilcoxon-signed-ranks test was used for the comparison of the mean folate values before and after UV exposures. A significant p-value was considered 0.05.

## Results

Twenty patients (10 men and 10 women) were included. The mean age was 35.8±14.16 years (range 11-56). Baseline characteristics of patients are shown in table 1. After 30 NB-UVB sessions, the mean serum folate level decrease from 2.76±0.59 ng/ml at baseline to 1.34±0.15 ng/ml (P=0.001, mean NB-UVB cumulative dose 40.33±16.86 J/cm^2^). Comparison of folate levels before and after phototherapy were also performed based on the sex, age and disease (table 2). Mean serum folate level significantly decreased in psoriasis and vitiligo patients; however, in patients with MF, reduction of folate status was not significant (table 2). 

Folate level changed before and after phototherapy was significantly observed in male and female groups and also patients under and above 40 years old (table 2). Otherwise, changes in serum folate levels were not significantly associated with cumulative UVB doses (r=-0.19, P=0.618). 

**Table 1:** Baseline characteristics of patients

**Parameters **	**Men**	**Women**	**Total**
No.(%) of patients	10(50)	10 (50)	20(100)
Age, y, )mean±SD(	34.2±15.08	37.4±13.78	35.8±14.16
Disease classification			
Psoriasis Vitiligo MF	6 2 2	4 5 1	10 7 3
Baseline serum folate (ng/ml); )mean±SE(	3.24±1.04	2.27±0.62	2.76±0.59
Mean cumulative NB-UVB doses (J/cm^2^) ;(mean±SD(	38.78±18.86	41.88±15.32	40.33±16.80

SD, standard deviation; SE, standard error; MF, mycosis fungoides.

**Table 2:** Effect of NB-UVB phototherapy on serum folate in patients based on sex, age and type of skin disease

**parameters**	**No**	**Serum folate (ng/ml)(mean±SE)**		**pvalue**
		**Before exposure**	**After exposure**	
Sex				
Male Female	10 10	3.24±1.04 2.27±0.62	1.52±0.25 1.15±0.51	0.005 0.005
Age, y				
40 ≥40	12 8	3.62±0.90 1.46±0.33	1.63±0.20 0.89±0.13	0.002 0.012
Disease classification Psoriasis Vitiligo MF	10 7 3	3.64±1.09 2.19±0.50 1.15±0.35	1.52±0.24 1.30±0.22 0.81±0.18	0.005 0.018 0.109
Total	20	2.76±0.59	1.34±0.15	0.001

*SE*, standard error; *MF*, mycosis fungoides.

## Discussion

Data of current study showed a statistically significant decrease in folate serum level in male and female, psoriasis and vitiligo patients after 30 exposures of NB-UVB radiation. Folate reduction after NB-UVB phototherapy have been previously reported by Shaheen et al (5) in vitiligo patients and by Elsaie et al (6) in psoriatic patients. However, the Rose et al.’s (7) study on 35 patients with psoriasis and the Cicarma et al.’s (8) study on 19 dermatologic patients did not find any significant difference in serum folate status before and after exposure to NB-UVB. Results obtained by Lajevardi et al. (9) also showed no significant changes. The different results of foregoing studies can be explained by the lower total cumulative dose used in these studies in comparison to our study. Furthermore, in our study, some patients achieved folate deficiency levels (2.5 ng/ml). Folate is the key factor in homocysteine detoxication. Inadequate folate status may result in hyperhomocysteinemia, an important risk factor for atherosclerotic vascular disease, changes in DNA that may eventuate in procarcinogenic consequences and raised risk for cognitive dysfunction (16). Hyperhomocysteinemia plays a role in the origin of at least some parts of NTDs (17). Patients with psoriasis may be deficient in folate and have a tendency to hyperhomocyteinemia that may predispose one to higher cardiovascular risk and other related complications (18). An association between vitiligo and reduced serum level of folic acid has been found (15). Photo degradation of folate may be more prominent in patients with folate deficiency (8). In our study, phototherapy aggravated the folate deficiency. Thus, evaluation of folate status in vitiligo and psoriatic patients who receive NB-UVB phototherapy before starting and during their phototherapy course and correction of any deficiencies should be considered as recommended by Elsaie et al (6). 

NB-UVB is generally advised by the dermatology community as a safe method for pregnant women (3). However our results indicated that a reduction in serum folate level is anticipated in patients on long-term phototherapy. Since inadequate folate status is associated with an increased risk for neural tube defects (17) and evidently periconceptional folic acid supplementation can prevent the major proportion of NTDs (19); therefore, folic acid supplements should be recommended in female patients of child bearing age who receive NB-UVB phototherapy.

Although we did not assess the influence of UVB phototherapy on folate level through photodegradation, but our study revealed that prolonged NB-UVB phototherapy significantly decrease serum folate level. Therefore, we suggest the evaluation of folate level in patients undergoing NB-UVB phototherapy at the beginning of the therapy and at intervals during their phototherapy course.

The limitation of the current study is the small number of patients who agreed to participate in the study.
